# Psychosocial determinants of the intention and self-efficacy to attend antenatal appointments among pregnant adolescents and young women in Cape Town, South Africa: a cross-sectional study

**DOI:** 10.1186/s12889-022-14138-0

**Published:** 2022-09-23

**Authors:** Ronel Sewpaul, Rik Crutzen, Priscilla Reddy

**Affiliations:** 1 Department of Health Promotion, Maastricht University/CAPHRI, P.O. Box 616, 6200 MD Maastricht, The Netherlands; 2 Health & Wellbeing, Human and Social Capabilities Division, Human Sciences Research Council, 118 Buitengraght Street, Cape Town, 8000 South Africa; 3 College of Health Sciences, University of KwaZulu-Natal, Durban, South Africa

**Keywords:** Intention, Self-efficacy, Psychosocial determinants, Antenatal care, Appointment keeping, Confidence interval-based estimation of relevance (CIBER), Adolescents, South Africa

## Abstract

**Background:**

Antenatal care is imperative for adolescents and young women, due to their increased risk of pregnancy-related complications. Evidence on the psychosocial determinants of antenatal attendance among this vulnerable group is lacking. This study assessed the relevance of the psychosocial sub-determinants of intention and self-efficacy to attend antenatal appointments among pregnant adolescents and young women in Cape Town, South Africa; with a view to informing behaviour change interventions.

**Methods:**

Pregnant women and girls aged 13-20 years were recruited to complete a cross-sectional questionnaire assessing their pregnancy experiences, pregnancy-related knowledge and psychosocial determinants related to antenatal care seeking. Confidence Interval Based Estimation of Relevance (CIBER) analysis was used to examine the association of the psychosocial sub-determinants with the intention and self-efficacy to attend antenatal appointments, and to establish their relevance for behaviour change interventions. The psychosocial sub-determinants comprised knowledge, risk perceptions, and peer, partner, family and individual participant attitudes.

**Results:**

The mean gestation age of participants (n=575) was 18.7 weeks, and the mean age was 18 years. Risk perceptions of experiencing preeclampsia and heavy bleeding during pregnancy or childbirth if clinic appointments are not attended had moderate mean scores and were positively correlated with intention and self-efficacy, which makes them relevant intervention targets. Several family, peer, partner and individual participant attitudes that affirmed timely appointment attendance had strong positive associations with intention and self-efficacy but their mean score were already high.

**Conclusions:**

Given the high means of the family, peer, partner and individual participant attitudes, the relevance of these attitudinal items as intervention targets was relatively low. Further studies are recommended to assess the relevance of these sub-determinants in similar populations.

**Supplementary Information:**

The online version contains supplementary material available at 10.1186/s12889-022-14138-0.

## Introduction

Antenatal care (ANC) is particularly important for adolescents and young women, due to their increased risk of pregnancy related complications and higher maternal and infant mortality rates [1–5]. It is therefore crucial that complications be identified or prevented early in an adolescent or young woman’s pregnancy and the necessary monitoring continued, through early and routine ANC.

Antenatal care can be defined as the care provided by skilled health-care professionals to pregnant women and girls to ensure the best health conditions for both mother and baby during pregnancy. Timely ANC enables early risk identification; prevention and management of pregnancy-related or concurrent diseases, and the provision of pregnancy-related health education and health promotion [6]. Previous studies have found associations between antenatal care and pregnancy outcomes [7–9]. In South Africa, basic antenatal care (BANC) is provided free of charge at public health facilities. The South African Guidelines for Maternity Care advise that women start receiving ANC in their first trimester [10]. Over 93% of pregnant women in South Africa receive some ANC. However, only 47% start receiving ANC in the first trimester [11] and 32% present late for their first ANC booking, that is, after 20 weeks [12]. In addition, only 75% attended the World Health Organisation (WHO) recommended minimum of at least four ANC appointments [11].

Timely initiation and routine attendance of ANC in Sub-Saharan Africa tends to be lower among adolescents compared to older women [13]. Furthermore, women with unintended pregnancies, which are highly prevalent among adolescents, are less likely to receive appropriate maternal healthcare. A national household survey found that 77% of pregnant adolescents in South Africa reported attending the requisite of at least four ANC visits, which was similar to that of all pregnant women [14]. Local clinic-based studies found lower ANC attendance among adolescents and very young women than for older women [9, 15]. Furthermore, over 18% of pregnant adolescents and young women in South Africa are HIV-positive [16]. Timely ANC facilitates early HIV diagnoses, and initiation of antiretroviral therapy and interventions to prevent mother-to-child transmission for the unborn baby.

The adolescent fertility rate in South Africa is 68 births per 1000 girls aged 15-19 years, which is over four times that of high-income countries [17]. The institutional maternal mortality ratio for adolescents in 2014-2016 was 76.9 deaths per 100,000 live births. Over 72% of the deaths among adolescent mothers were from factors including non-pregnancy-related infections (HIV/AIDS-related, tuberculosis, or pneumonia), hypertension, obstetric haemorrhage, and medical and surgical disorders [18]; factors that can be managed through timely ANC. Given the higher risks of pregnancy-related complications among adolescents and young women and their suboptimal utilization of ANC, it is important to understand the determinants of ANC attendance behaviour among this vulnerable group.

Factors affecting delay and frequency of ANC access among adolescents in South Africa include both health systems-level factors such as interactions with health care providers, long wait times, comfort level of the facility and the quality of health education and support received for childbirth and parenting; as well as individual-level factors such as lack of knowledge regarding the importance of timely ANC attendance, support by the partner/boyfriend, pregnancy before marriage being regarded as socially deviant, financial barriers, distance travelled to access ANC services, HIV status, and fear and stigma about disclosing their pregnancies [19–22]. Lack of autonomy to make healthcare decisions, education levels, urban vs rural residence, parity, and cultural norms are further contributing factors identified in other countries [23].

Social cognitive theories, such as the Theory of Planned Behaviour [24] and the Reasoned Action Approach (RAA) [25] enable an understanding of the (sub-) determinants of a behaviour in order to develop interventions to improve the behaviour; in this case; antenatal appointment attendance. The RAA posits that intention is the most immediate determinant of performing a behaviour. Intention is predicted by sub-determinants, including attitudes about the behaviour, subjective norms, and perceived control over performing the behaviour. Perceived behavioural control is measured by self-efficacy. Other sub-determinants include beliefs, knowledge about the behaviour, and risk perceptions.

There is a lack of information on the psychosocial determinants of antenatal appointment attendance behaviour in adolescents and young women. Using a Confidence Interval Based Estimation of Relevance (CIBER) analysis approach [26], this study assesses the associations of risk perceptions, social support, individual attitudes, and peer, family and partner attitudes regarding ANC attendance with the self-efficacy and intention to attend antenatal appointments among pregnant adolescents and young women in Cape Town, South Africa. It seeks to identify the most relevant sub-determinants to target in interventions designed to improve antenatal appointment attendance.

## Methods

### Study design and setting

The current study analyses are part of the study titled “A Pilot Study of Improving Outcomes in Teenage Pregnancy Using a Combined Tailored M- Health Program and Motivational Interviewing Intervention” with trial registration number PACTR201912734889796. In this study, pregnant adolescents and young women were recruited to be enrolled in a pilot randomised controlled trial (RCT) that tested a behavioural intervention to improve their health care seeking and general health behaviours during pregnancy. Data were collected at baseline upon being recruited into the study as well as at follow-up after giving birth. This study reports on the baseline data which was collected during May – December 2018. A cross-sectional study design was used in the baseline survey. The study follows the STROBE Statement for reporting observational studies [27]. A sample of 200 (100 participants per group) was decided upon for the pilot RCT. However, given the high expected drop-out rate in adolescent public health longitudinal studies and that participants with missing information on contact details and pregnancy characteristics would be excluded from registration onto the mobile intervention, it was decided to recruit three times the planned sample size for the baseline survey.

In the South African primary healthcare system, which serves the majority of the population, pregnant girls and women receive antenatal care and maternity services at outpatient clinics, community health centres (CHC) or Midwife Obstetric Units (MOUs). The study was conducted in Cape Town in the Western Cape province of South Africa, which is predominantly urban. In 2019/20, 9.5% of the 67 485 in-facility deliveries in Cape Town were among adolescents aged 10-19 years. This was slightly lower than the 13.2% national adolescent in-facility delivery rate [12].

### Recruitment of participants

Pregnant women and girls aged 13-20 years were eligible to be included and were recruited to participate in this study. Recruited women and girls who did not consent to participation were excluded from the study. Discussions were held with the Western Cape Provincial Department of Health to identify priority areas and clinics from which to recruit pregnant girls and young women. Based on these discussions, 16 community facilities that provided ANC (comprising public health clinics, CHCs and MOUs) were identified from which to recruit participants. These facilities were located in four of the eight health sub-districts in Cape Town; namely, Cape Town Eastern, Cape Town Northern, Mitchells Plain and Tygerberg. Participants were recruited while attending ANC at the facilities. Facility managers were contacted to inform them about the study and to engage them in discussions about recruitment and data collection activities. Researchers introduced the study to the ANC attendees in the waiting areas. In some cases, facility staff referred the researchers to groups of potential participants. Participants were also recruited from communities through social networks. The research assistants explained the study to potential participants in their language of choice. The research assistants were fluent in English, and either Afrikaans or isiXhosa, which are the three predominant official languages spoken in Cape Town.

### Questionnaire development and data collection

Questionnaire development was guided by the RAA [25] and the I-Change model for understanding health behaviour [28]. The questionnaire items were informed by a literature review that identified psychosocial and socioeconomic factors associated with ANC attendance behaviours in young women and adolescents. The key thematic areas in the questionnaire were demographic characteristics, previous pregnancies, mental health status, knowledge of HIV and TB, knowledge regarding appointment attendance, risk perceptions; peer, partner and family support and attitudes regarding appointment attendance, and participant attitudes, self-efficacy and intention towards attending ANC appointments. The questionnaire was developed in English and then translated and back-translated into Afrikaans and isiXhosa by post graduate students proficient in each language who were working as part of the study’s research term.

Twenty research assistants were trained in recruitment and data collection activities and were selected to work in the study. Participants completed a self-administered structured questionnaire on an electronic tablet or mobile phone. The interviews were facilitated by the research staff. In a few cases where the participant was not comfortable with completing the questionnaire themselves, the research assistant administered the questionnaire. While the questionnaire was available to complete in Afrikaans, isiXhosa and English, only two participants opted to answer the questionnaire in Afrikaans and none in isiXhosa. Participants received a R50 (approx. $3) incentive upon completion of the questionnaire. The questionnaire took on average 60 minutes to complete.

### Measures

The two dependent variables in this study were intention and self-efficacy to attend ANC appointments. Intention was measured by the item “I intend to attend all the clinic appointments” and self-efficacy was measured by the item “I am confident in my ability to attend clinic appointments, when I am feeling lazy and tired”. Both items were measured on a 4-point Likert scale, where 1=Strongly disagree, 2=Disagree, 3=Agree, and 4=Strongly agree. Hence, higher scores on the items indicated higher intention and self-efficacy to attend appointments.

The independent variables were classified into six groups i) risk perceptions, ii) social support from family, friends and partners for attending ANC, iii) partner attitudes regarding ANC, iv) peer attitudes and norms regarding ANC, v) family attitudes regarding ANC, and vi) participant attitudes regarding attending ANC. Seven items assessed risk perceptions regarding the implications of not attending or missing ANC appointments and the risks of pregnancy complications, with response options 1=Strongly disagree, 2=Disagree, 3=I don’t know, 4=Agree and 5=Strongly agree. Social support for attending ANC was assessed by three items regarding the encouragement received from each of family, friends and partner/boyfriend to attend ANC appointments, and response options were 1=Strongly disagree, 2=Disagree, 3=Agree, and 4=Strongly agree. Four items assessed partner/boyfriend attitudes regarding ANC attendance. Five items assessed the attitudes regarding ANC attendance among the participants’ friends who were or had been pregnant and one item assessed the norms regarding ANC attendance among the participants’ friends who were or had been pregnant. Response options for partner attitudes, peer norms and peer attitudes were 1=Strongly disagree, 2=Disagree, 3=Agree, and 4=Strongly agree. Participants who did not have a partner/boyfriend or did not have friends who had been pregnant did not answer the respective questions. Seven items assessed family attitudes regarding ANC attendance with response options 1=Strongly disagree, 2=Disagree, 3=I don’t know, 4=Agree and 5=Strongly agree. Thirteen items assessed participants’ attitudes regarding attending ANC, with response options 1=Strongly disagree, 2=Disagree, 3=I don’t know, 4=Agree and 5=Strongly agree. Therefore, the risk perception, family attitude and participant attitude items were assessed on a 5-point Likert scale while the social support, partner attitude and peer attitude and norm items were assessed on a 4-point Likert scale. The individual sub-determinant items included in the study are presented in Additional file 1.

Sociodemographic characteristics of the participants included date of birth, estimated date of delivery (EDD), estimated last menstrual date (or month), population group, type of residence, school or college attendance and previous pregnancies. Gestational age (number of weeks pregnant) was calculated using the EDD. When the participant did not know their EDD the last menstrual date was used instead. Age was calculated from the date of birth.

### Statistical analysis

Data analyses were conducted using R version 4.0.3 and the Statistical Package for Social Sciences (SPSS) version 27. Data was collected from 615 participants, of which 575 (93.5%) answered the questions on intention and self-efficacy. Descriptive statistics of the sociodemographic characteristics were presented as means for interval variables and proportions for nominal variables. Confidence Interval Based Estimation of Relevance (CIBER) analysis [26] was conducted to assess the relevance of the psychosocial sub(determinants) (knowledge, risk perception, social support; peer, family, and partner attitudes and participant attitudes) of the intention and self-efficacy to attend ANC appointments.

CIBER is a data visualization method that uses a diamond plot to assess the most relevant sub-determinants for intervention development. It visualises the mean of each sub-determinant, its correlation with one or more determinants, and the confidence intervals of both these estimates. The diamond plot is divided into a left- and right-hand panel with diamond shapes. The question that assessed each sub-determinant with its anchors (highest and lowest response options on the Likert scale) is shown on the left of the left-hand panel. Each diamond shape in the left panel shows the mean of each sub-determinant item and its 99.99% confidence interval. Diamond shapes facilitate representation of the mean and the confidence interval in one shape. Generally, the redder the diamonds are the lower the item means and the greener the diamonds are the higher the item means. The dots around the left-hand panel diamonds show all the participants’ item scores with jitter added to prevent overplotting.

Each diamond in the right panel shows the correlation between the sub-determinant items and the two dependent variables (self-efficacy and intention) with their 95% confidence intervals. Purple diamonds represent the correlations of the sub-determinants and self-efficacy to attend ANC appointments when feeling lazy and tired. Yellow diamonds represent the correlations of the sub-determinants and the intention to attend all the ANC appointments. The fill colour of the diamonds indicates the strengths and directions of association – the redder the fill colour of the diamonds are, the stronger and more negative the correlations are; the greener the diamonds are, the stronger and more positive the correlations are; and the greyer the diamonds are, the weaker the correlations are. At the top of the plot is the confidence interval of the explained variance (R^2^) of self-efficacy and intention based on all items included in the plot. A CIBER plot was produced for the items relating to knowledge, risk perception, social support, family attitudes, peer and partner attitudes, and participant attitudes. The combination of correlation coefficients, means, and their confidence intervals were then interpreted to identify the relevant items that could be targeted in an intervention. Items that have low or mid-level means in the undesirable direction and have strong associations with the determinants of intention and self-efficacy are considered relevant sub-determinants for intervening upon.

## Results

### Sociodemographic characteristics of the sample

Of the 575 participants, the mean gestation age (weeks pregnant) was 18.7 weeks and the mean age was 18 years (Table 1). The majority (73.3%) lived in formal dwellings such as brick houses and apartment blocks, while 23.6% lived in informal dwellings that included informal settlement houses and houses made of mud and tin. The majority of the participants classified themselves as ‘coloured’ (63.0%) and 36.3% classified themselves as black African. Almost two thirds of the participants were not currently attending an educational institution, 29.7% were attending school and 6.1% were attending a Further Education and Training (FET) college. The percentage of participants who reported that they had been pregnant previously was 11.1% and 13.8% reported that they had considered having an abortion. The mean scores for self-efficacy to attend ANC appointments when feeling lazy or tired and for intention to attend all ANC appointments were relatively high, with participants scoring an average of 3.3 and 3.4 respectively on the scale from 1 to 4.

**Table 1:** Description of the sample (*n*=575)

	Number (%)
Age (years) (Mean, S.D.)	18.0 (1.6)
13-15	45 (7.8)
16-17	146 (25.4)
18-20	384 (66.8)
Gestational (Mean, S.D.)	18.7 (6.5)
<= 12 weeks	119 (21.1)
13-24 weeks	342 (60.6)
25-41 weeks	103 (18.3)
Population group	
Black African	208 (36.3)
‘coloured'	361 (63.0)
White	1 (0.2)
Indian/Asian	2 (0.3)
Other	1 (0.2)
Type of dwelling	
Formal dwelling	418 (73.3)
Informal dwelling	134 (23.6)
Other	18 (3.2)
Attending an educational institution	
Attend school	171 (29.7)
Attend an FET college	35 (6.1)
Not attending	369 (64.2)
Had previously been pregnant	
Yes	64 (11.1)
No	511 (88.9)
Considered having an abortion	
Yes	79 (13.8)
No	492 (86.1)
Self efficacy: I am confident in my ability to attend clinic appointments, when I am feeling lazy and tired (Mean, S.D.)	3.3 (0.7)
Intention: I intend to attend ALL the clinic appointments (Mean, S.D.)	3.4 (0.6)

*S.D.* Standard deviation, *FET* Further Education and Training college. Refers to colleges offering vocational courses

### CIBER Plot

**Fig. 1.**
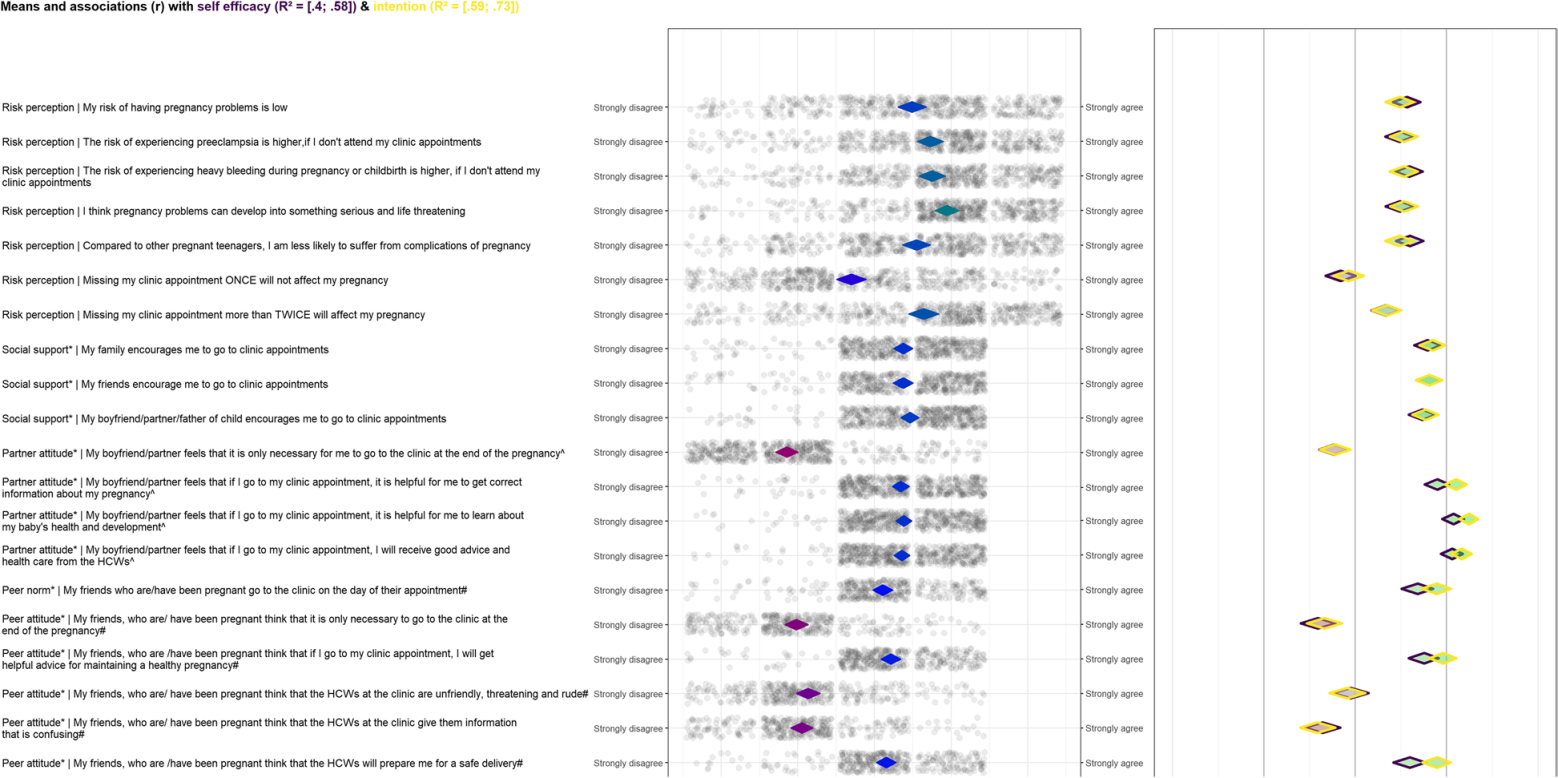

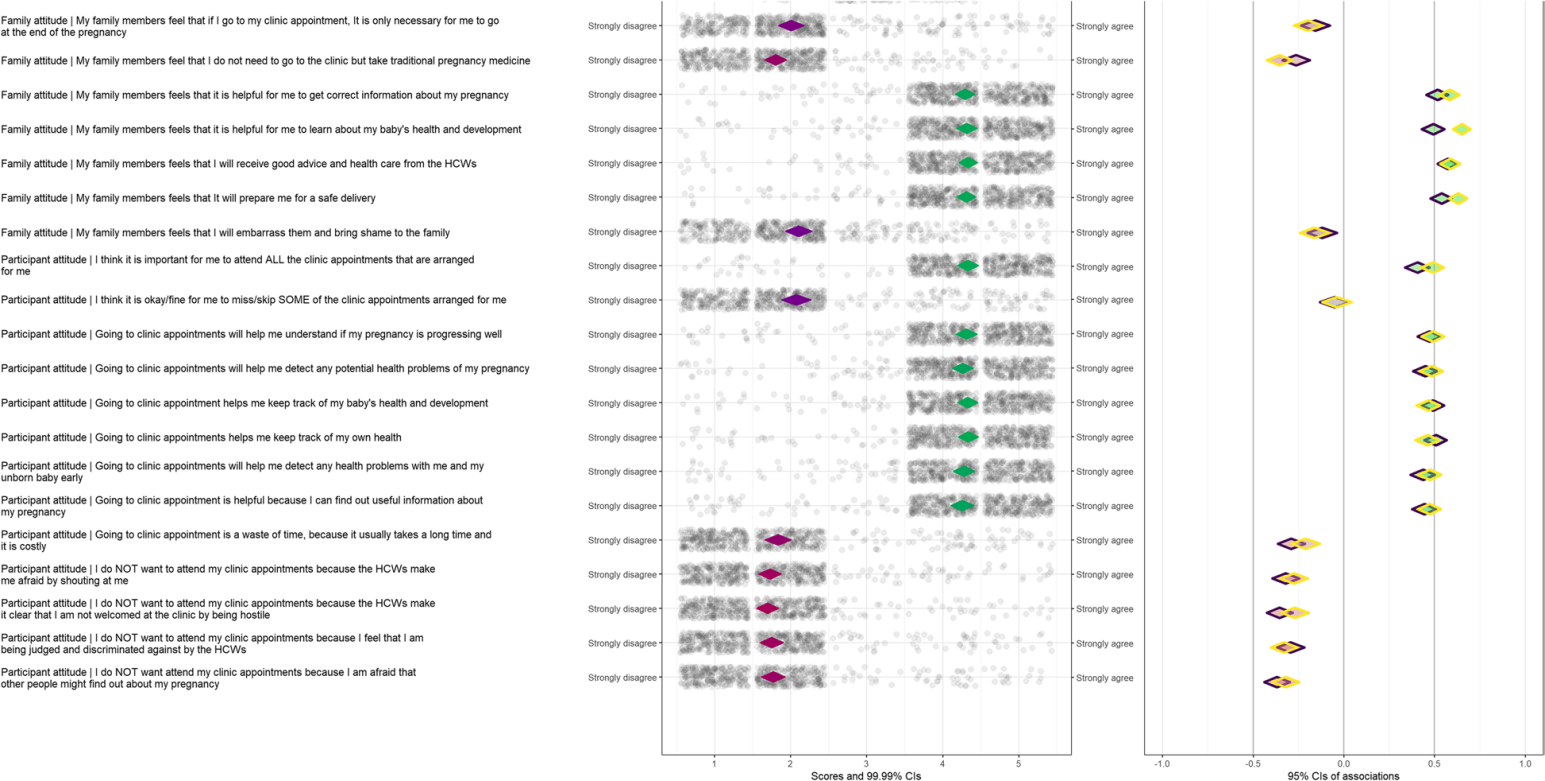
Confidence Interval Based Estimation of Relevance (CIBER) plot showing the mean scores of psychosocial sub-determinants (knowledge, risk perception, social support; peer, family, and partner attitudes and participant attitudes) and their associations with the intention and self-efficacy to attend antenatal appointments

The CIBER plot is presented in Fig. 1. The sub-determinant items collectively explained 40% to 58% of the variance in self-efficacy to attend ANC appointments when feeling lazy and tired, and 59% to 73% of the variance in intention to attend all ANC appointments.

### Risk perceptions

The risk perception items that were positively correlated with self-efficacy and intention were “My risk of having pregnancy problems is low”, “The risk of experiencing preeclampsia is higher if I don’t attend my clinic appointments”, The risk of experiencing heavy bleeding during pregnancy or childbirth is higher, if I don’t attend my clinic appointments”, “I think pregnancy problems can develop into something serious and life threatening”, “Compared to other pregnant teenagers, I am less likely to suffer from complications of pregnancy” and “Missing my clinic appointment more than TWICE will affect my pregnancy”. The perception that pregnancy problems can develop into something serious and life threatening had a high mean score meaning that it was frequently reported and in the desirable direction. Therefore, it would not be prioritised for intervention development. The remaining five risk perceptions had mid to upper level means and could therefore be considered as relevant sub-determinants for intervening upon. The item “Missing my clinic appointment ONCE will not affect my pregnancy” had a low mean score and was not correlated with the dependent variables.

### Social support from family, friends, and partners

The three social support items, that is, “My family encourages me to go to clinic appointments”, “My friends encourage me to go to clinic appointments” and “My boyfriend/partner/father of child encourages me to go to clinic appointments” had high mean scores and positive correlations with self-efficacy and intention. The high mean scores show that many participants reported encouragement from their family, friends and partners. Therefore, these items would not be prioritised for intervention development.

### Peer attitudes and norms

Mean scores for all five items on peer attitudes were in the desirable direction. Attitudes of the participants’ friends who are/have been pregnant that ANC attendance provides them with helpful pregnancy advice and can prepare them for safe deliveries; and the norm that the participants’ friends attend their appointments were all high and were positively correlated with self-efficacy and intention. These peer attitudes showed stronger correlations with intention than with self-efficacy. The means for peer attitudes that were disapproving of ANC attendance, that is, friends who have been pregnant feeling that it is only necessary to go to the clinic at the end of the pregnancy, friends feeling that the health care workers give them confusing information, and friends feeling that the health care workers are unfriendly, threatening and rude, were all low and had relatively weak negative correlations with self-efficacy and intention.

### Partner attitudes

All three partner/boyfriend attitudes that affirmed ANC attendance, namely, “My boyfriend/partner feels that if I go to my clinic appointment, it is helpful for me to get correct information about my pregnancy”, “My boyfriend/partner feels that if I go to my clinic appointment, it is helpful for me to learn about my baby’s health and development”, and “My boyfriend/partner feels that if I go to my clinic appointment, I will receive good advice and health care from the health care workers” had high mean scores and were therefore in the desirable direction. These items also had positive correlations with self-efficacy and intention, that were stronger than those for peer attitudes and norms. The mean for the partner attitude that was disapproving of ANC attendance, namely, “My boyfriend/partner feels that it is only necessary to for me to go to the clinic at the end of the pregnancy” was low and had a weak negative association with self-efficacy and intention. The partner attitudes that affirmed ANC attendance showed stronger correlations with intention than with self-efficacy.

### Family attitudes

The four items on family members’ positive attitudes on ANC attendance, namely “My family members feel that it is helpful for me to get correct information about my pregnancy”, “My family members feel that it is helpful for me to learn about my baby’s health and development”, “My family members feel that I will receive good advice and health care from the health care workers” and “My family members feel that it will prepare me for a safe delivery”, had high means in the desirable direction and strong positive correlations with self-efficacy and intention. Similarly, family attitudes against timely ANC attendance, namely, “My family members feel that if I go to my clinic appointment, it is only necessary for me to go at the end of the pregnancy”, “My family members feel that I do not need to go to the clinic but take traditional pregnancy medication” and “My family members feel that I will embarrass them and bring shame to the family” were negatively correlated with self-efficacy and intention but had low mean scores. This means that few participants reported that their families had these adverse attitudes. Notably, many family attitude items had stronger correlations with intention than with self-efficacy.

### Participant attitudes

The following participant attitude items had high mean scores in the desirable direction and strong positive correlations with self-efficacy and intention : “I think it is important for me to attend all the clinic appointments that are arranged for me”, “Going to clinic appointments will help me understand if my pregnancy is progressing well”, “Going to clinic appointments will help me detect any potential health problems of my pregnancy”, “Going to clinic appointment helps me keep track of my baby’s health and development”, “Going to clinic appointments helps me keep track of my own health”, “Going to clinic appointments will help me detect any health problems with me and my unborn baby early”, and “Going to clinic appointments is helpful because I can find out useful information about my pregnancy”. The following items had low mean scores and were negatively correlated with self-efficacy and intention: “Going to clinic appointment is a waste of time, because it usually takes a long time and it is costly”, “I do NOT want to attend my clinic appointments because the health care workers make me afraid by shouting at me”, “I do NOT want to attend my clinic appointments because the health care workers make it clear that I am not welcomed at the clinic by being hostile”, “I do NOT want to attend my clinic appointments because I feel that I am being judged and discriminated against by the health care workers” and “I do NOT want attend my clinic appointments because I am afraid that other people might find out about my pregnancy”. The attitude that it was fine to miss some of the scheduled ANC appointments had very weak correlations with the dependent variables. While the attitude items had strong associations with self-efficacy and intention, they were in the desirable direction and few participants exhibited adverse attitudes toward attending appointments.

## Discussion

The study identified several risk perception, social support and attitudinal sub-determinants that were associated with self-efficacy and intention to attend ANC appointments among pregnant adolescents and young women in Cape Town, South Africa. The sub-determinants varied in their relevance for intervention development.

The study found that self-reported intention and self-efficacy to attend appointments were already very high. Participants had high mean scores of 3.3 and 3.4 out of 4 for self-efficacy and intention respectively. This meant that the majority of participants agreed or strongly agreed with the statements on self-efficacy and intention to attend appointments, thereby suggesting little room for improvement. But, data suggests that ANC attendance remains a public health problem. In Cape Town, 32% of pregnant women book late for their first ANC visit [12] but the rate for adolescents is unknown. In South Africa, only 77% of adolescents attend the requisite of at least four ANC visits [14]. Even if the ANC attendance for adolescents and adults were similar, then a quarter of adolescents attend less than four ANC visits and almost a third present late for their first ANC booking. This suggests discrepancy between the actual behaviour and the reported intention and self-efficacy rates found in this study.

The intention-behaviour gap highlights that individuals may have strong intentions to practise a behaviour and actual control over practising it, but may not practise the behaviour [29], as other factors can contribute to the behavioural outcome. In such cases, the likelihood of practising the behaviour can be strengthened when participants are asked to develop an implementation intention or a plan about how and when they will practise the behaviour [25]. In addition, the high intention and self-efficacy could be due to social desirability bias in reporting. Non-attendance of ANC appointments is perceived as a deviant health behaviour. Therefore, the participants may have been more likely to report the more socially acceptable intentions. We tried to minimise this bias through the use of self-administered questionnaires and informed consent of the confidentiality of study procedures. It can be argued that high self-reported intentions and self-efficacy could be due to selection bias of participants who had better health seeking behaviour. However, all participants at the clinics and in communities who were eligible to participate were approached and invited to participate in the study and an incentive was provided to all participants regardless of their responses.

The two risk perception items on the perceived risk of experiencing preeclampsia and heavy bleeding during pregnancy or childbirth if clinic appointments are not attended had mid-level mean scores and were positively correlated with intention and self-efficacy. Participants had lower scores for the risk perceptions than for social support and family, peer, partner and participant attitudes, suggesting scope for improving knowledge of these items among young pregnant women. Interventions that educate pregnant adolescents and women about the consequences of irregular and late ANC and about health complications that can develop during pregnancy will create realistic risk perceptions.

The risk perception items had positive but weaker correlations with intention and self-efficacy than for the social support and attitude items. Therefore, improving risk perceptions will likely lead to smaller improvements in appointment attendance than improving higher-order sub-determinants like attitudes. However, changeability is important to consider when selecting determinants for intervention development [30]. Creating realistic risk perceptions about a behaviour can be relatively easier to induce than changing attitudes towards that same behaviour. For this reason, the findings should be interpreted alongside behaviour change expertise to select determinants that are both relevant and changeable and to translate them into practical applications.

The associations between sub-determinants and outcomes are not necessarily unidirectional. In some cases, there can be a reciprocal relationship. An example of this is the risk perception items “My risk of having pregnancy problems is low” and “Compared to other pregnant teenagers, I am less likely to suffer from complications of pregnancy”. Participants had moderate scores for these items, and they were positively correlated with self-efficacy and intention. However, low risk perceptions could be reflective of antenatal attendance that was already high at the time of data collection. Longitudinal data are required to test these reciprocal relationships.

While the identified attitudes and social support items were strongly associated with intention and self-efficacy, their mean scores imply that their relevance and prioritization for behaviour change interventions is low. The reason for this is that, for the majority of the items on social support, participant attitudes and peer, partner and family attitudes that were correlated with intention and self-efficacy, the mean scores were already in the desirable direction. That is, positive sub-determinants about appointment attendance had high mean scores, with participants largely in agreement with these statements. Similarly, negative or discouraging sub-determinants about appointment attendance had low mean scores, with participants largely disagreeing with these statements. Therefore, while they are identified sub-determinants, interventions addressing them would be addressing the relatively small numbers of participants who exhibit the unfavourable attitudes and perceptions. If these sub-determinants are targeted in an intervention, this would mean that these items need to be reinforced. Alternatively, the intervention messages could be tailored to only the small subgroup of participants and their families and partners who do not view timely ANC as beneficial.

Family and partner attitudes exhibited very strong associations with intention and self-efficacy, and these associations were stronger than for peer attitudes. Other studies have identified the support and positive opinions from family and partners as influencing pregnant women’s health seeking behaviour [31, 32]. The positive attitudes of family members, partners and friends can normalise regular ANC attendance. Adolescents can be more vulnerable to external influences and hence, the support from their social groups would assist them to make autonomous decisions about their health care seeking. Community level and individual interventions can improve family and partner attitudes to ANC by involving them in the interventions. Interventions that involve expectant fathers in maternal health care have been linked to improved health care seeking [33, 34].

The study has some limitations. Firstly, its cross-sectional nature limits causal interpretations but indicates associations between psychosocial sub-determinants and self-efficacy and intention. Secondly, the response options for the sub-determinants were measured on different scales. The risk perceptions, participant attitudes, and family attitudes were measured on a 5-point scale (strongly agree, agree, I don’t know, disagree and strongly disagree); whereas the partner and peer attitudes were measured on a 4-point scale (strongly agree, agree, disagree and strongly disagree). The 4-point scale excluded an option for participants who were unsure or had neutral responses, thus excluding their ability to take a neutral stance. The CIBER plot allowed for the sub-determinants to be plotted together.

## Conclusions

This pioneering study provides information on the psychosocial sub-determinants of self-efficacy and intention to attend ANC appointments, and their relevance, among pregnant adolescents and young women in a South African context. The findings can contribute to improving health systems by targeting individual-level behaviour change. These findings can be considered by policy makers, health systems programmers and behaviour change specialists to inform interventions to enhance ANC uptake among pregnant youth. The study found that risk perceptions of experiencing preeclampsia and heavy bleeding during pregnancy or childbirth if clinic appointments are not attended were relevant intervention targets because they were positively correlated with intention and self-efficacy and showed moderate mean scores. Family, peer, partner and individual participant attitudes that affirmed timely appointment attendance showed strong positive associations with intention and self-efficacy. However, due to their high mean scores, the relevance of these attitudinal items in interventions to enhance ANC uptake in this study population is considered low. It is recommended to replicate this study in other provinces in South Africa and in rural settings to identify relevant psychosocial determinants in these areas.

## Supplementary Information

**Additional file 1.**

## Data Availability

The datasets used and/or analysed during the current study available from the corresponding author on reasonable request.

## Abbreviations

CIBER: Confidence interval-based estimation of relevance
ANC: Antenatal care
BANC: Basic antenatal care
WHO: World Health Organisation
RCT: Randomised controlled trial
CHC: Community health centre
MOU: Midwife Obstetric Units
RAA: Reasoned Action Approach (a cognitive behavioural theory)
EDD: Estimated date of delivery for a pregnant woman
SPSS: Statistical Package for Social Sciences
FET: Further Education and Training
