# Supplementary material for: Psychosocial determinants of the intention and self-efficacy to attend antenatal appointments among pregnant adolescents and young women in Cape Town, South Africa: a cross-sectional study
Source: BMC Public Health. 2022 Sep 23;22:1809. doi: 10.1186/s12889-022-14138-0 (PMC9502574; doi:10.1186/s12889-022-14138-0)
Supplement: Supplementary file 1 — Additional file 1. [file 12889_2022_14138_MOESM1_ESM.docx]

**Risk perceptions**

This section is about your perceptions on the clinic visits. Please answer to the best of your ability. Please select your response.

|  | Strongly Disagree (1) | Disagree (2) | I don’t know (3) | Agree (4) | Strongly agree (5) |
| --- | --- | --- | --- | --- | --- |
| 1 My risk of having pregnancy problems is low |  |  |  |  |  |
| 2 The risk of experiencing preeclampsia (e.g. high blood pressure) is higher, if I don’t attend my clinic appointments |  |  |  |  |  |
| 3 The risk of experiencing heavy bleeding during pregnancy or childbirth is higher, if I don’t attend my clinic appointments |  |  |  |  |  |
| 4 I think pregnancy problems can develop into something serious and life threatening |  |  |  |  |  |
| 5 Compared to other pregnant teenagers, I am less likely to suffer from complications of pregnancy (e.g. hypertension, preeclampsia, or heavy bleeding during childbirth) |  |  |  |  |  |
| 6 Missing my clinic appointment ONCE will not affect my pregnancy |  |  |  |  |  |
| 7 Missing my clinic appointment more than TWICE will affect my pregnancy |  |  |  |  |  |

Participant attitudes

This section is to understand your attitudes about going to clinic appointments. Please answer to the best of your ability. Please select your response.

|  | Strongly Disagree (1) | Disagree (2) | I don’t know (3) | Agree (4) | Strongly agree (5) |
| --- | --- | --- | --- | --- | --- |
| 1 I think it is important for me to attend ALL the clinic appointments that are arranged for me |  |  |  |  |  |
| 2 I think it is okay/fine for me to miss/skip SOME of the clinic appointments that are arranged for me |  |  |  |  |  |
| 3 Going to clinic appointments will help me understand if my pregnancy is progressing well |  |  |  |  |  |
| 4 Going to clinic appointments will help me detect any potential health problems of my pregnancy |  |  |  |  |  |
| 5 Going to clinic appointment helps me keep track of my baby’s health and development |  |  |  |  |  |
| 6 Going to clinic appointments helps me keep track of my own health |  |  |  |  |  |
| 7 Going to clinic appointment will help me detect any health problems with me and my unborn baby early |  |  |  |  |  |
| 8 Going to clinic appointment is helpful because I can find out useful information about my pregnancy |  |  |  |  |  |
| 9 Going to clinic appointment is a waste of time, because it usually takes a long time and it is costly. |  |  |  |  |  |
| 10 I do NOT want to attend my clinic appointments because the health care workers make me afraid by shouting at me |  |  |  |  |  |
| 11 I do NOT want to attend my clinic appointments because the health care workers make it clear that I am not welcomed at the clinic by being hostile |  |  |  |  |  |
| 12 I do NOT want to attend my clinic appointments because I feel that I am being judged and discriminated against by the health care workers |  |  |  |  |  |
| 13 I do NOT want to attend my clinic appointments because I am afraid that other people might find out about my pregnancy |  |  |  |  |  |

**Social support**

This section is about the social support for your pregnancy. Please answer to the best of your ability. Please select your response

|  | Strongly disagree (1) | Disagree (2) | Agree (3) | Strongly agree (4) |
| --- | --- | --- | --- | --- |
| 1 My family encourages me to go to clinic appointments |  |  |  |  |
| 2 My friends encourage me to go to clinic appointments |  |  |  |  |
| 3 My boyfriend/partner/father of child encourages me to go to clinic appointments |  |  |  |  |

**Peer attitudes and norms ***

This section is about your friends’ behaviours and attitudes of pregnancy. Please answer to the best of your ability. If you do NOT have a friend who has been pregnant, please select 'not applicable'.

|  | Strongly disagree (1) | Disagree (2) | Agree (3) | Strongly Agree (4) |
| --- | --- | --- | --- | --- |
| 1 My friends who are/have been pregnant go to the clinic on the day of their appointment |  |  |  |  |
| 2 My friends, who are/ have been pregnant think that it is only necessary to go to the clinic at the end of the pregnancy |  |  |  |  |
| 3 My friends, who are /have been pregnant think that if I go to my clinic appointment, I will get helpful advice for maintaining a healthy pregnancy |  |  |  |  |
| 4 My friends, who are/ have been pregnant think that the health care workers at the clinic are unfriendly, threatening and rude |  |  |  |  |
| 5 My friends, who are/ have been pregnant think that the health care workers at the clinic give them information that is confusing |  |  |  |  |
| 6 My friends, who are /have been pregnant think that the health care workers will prepare me for a safe delivery |  |  |  |  |

* Item 1 refers to a peer norm. Items 2-6 refer to peer attitudes.

**Family attitudes**

This section is about your family members’ attitudes of your pregnancy. Please answer to the best of your ability. Please select your response.

|  | Strongly Disagree (1) | Disagree (2) | Agree (3) | Strongly Agree (4) | I don’t know (5) |
| --- | --- | --- | --- | --- | --- |
| 1 My family members feels that if I go to my clinic appointment, It is only necessary for me to go at the end of the pregnancy |  |  |  |  |  |
| 2 My family members feel that I do not need to go to the clinic but take traditional pregnancy medicine |  |  |  |  |  |
| 3 My family members feels that it is helpful for me to get correct information about my pregnancy |  |  |  |  |  |
| 4 My family members feels that it is helpful for me to learn about my baby’s health and development |  |  |  |  |  |
| 5 My family members feels that I will receive good advice and health care from the health care workers |  |  |  |  |  |
| 6 My family members feels that It will prepare me for a safe delivery |  |  |  |  |  |
| 7 My family members feels that I will embarrass them and bring shame to the family |  |  |  |  |  |

**Partner/boyfriend attitudes**

This section is about your partner/boyfriend’s attitudes of pregnancy. Please answer as honestly as you can, and select the number that is appropriate for you. If you do NOT have a partner/boyfriend, please circle “not applicable”.

|  | Strongly disagree (1) | Disagree (2) | Agree (3) | Strongly Agree (4) |
| --- | --- | --- | --- | --- |
| 1 My boyfriend/partner feels that it is only necessary for me to go to the clinic at the end of the pregnancy |  |  |  |  |
| 2 My boyfriend/partner feels that if I go to my clinic appointment, it is helpful for me to get correct information about my pregnancy |  |  |  |  |
| 3 My boyfriend/partner feels that if I go to my clinic appointment, it is helpful for me to learn about my baby’s health and development |  |  |  |  |
| 4 My boyfriend/partner feels that if I go to my clinic appointment, I will receive good advice and health care from the health care workers |  |  |  |  |
| 5 My boyfriend/partner feels that if I go for an HIV test that I don’t trust and love him |  |  |  |  |
| 6 My boyfriend/partner feels that if I have had an HIV test he does not have to have one |  |  |  |  |
| 7 My boyfriend/partner feels that since I am already pregnant, we don’t have to use a condom when we have sex |  |  |  |  |
| 8 Now that I am pregnant, I am worried that my partner/boyfriend will sleep with other girls |  |  |  |  |

**Self-efficacy**

This section is about your feelings of confidence in attending the clinic appointments. Please answer to the best of your ability. Please select your response.

|  | Strongly Disagree (1) | Disagree (2) | Agree (3) | Strongly Agree (4) | Not applicable (5) |
| --- | --- | --- | --- | --- | --- |
| I am confident in my ability to attend my clinic appointments, when I am feeling lazy and tired |  |  |  |  |  |

**Intention**

This section is about your intention related to clinic appointments. Please answer to the best of your ability. Please select your response.

|  | Strongly Disagree (1) | Disagree (2) | Agree (3) | Strongly Agree (4) |
| --- | --- | --- | --- | --- |
| I intend to attend ALL the clinic appointments |  |  |  |  |
